# Gene Coexpression Network Analysis as a Source of Functional Annotation for Rice Genes

**DOI:** 10.1371/journal.pone.0022196

**Published:** 2011-07-22

**Authors:** Kevin L. Childs, Rebecca M. Davidson, C. Robin Buell

**Affiliations:** Department of Plant Biology, Michigan State University, East Lansing, Michigan, United States of America; The University of Maryland, United States of America

## Abstract

With the existence of large publicly available plant gene expression data sets, many groups have undertaken data analyses to construct gene coexpression networks and functionally annotate genes. Often, a large compendium of unrelated or condition-independent expression data is used to construct gene networks. Condition-dependent expression experiments consisting of well-defined conditions/treatments have also been used to create coexpression networks to help examine particular biological processes. Gene networks derived from either condition-dependent or condition-independent data can be difficult to interpret if a large number of genes and connections are present. However, algorithms exist to identify modules of highly connected and biologically relevant genes within coexpression networks. In this study, we have used publicly available rice (*Oryza sativa*) gene expression data to create gene coexpression networks using both condition-dependent and condition-independent data and have identified gene modules within these networks using the Weighted Gene Coexpression Network Analysis method. We compared the number of genes assigned to modules and the biological interpretability of gene coexpression modules to assess the utility of condition-dependent and condition-independent gene coexpression networks. For the purpose of providing functional annotation to rice genes, we found that gene modules identified by coexpression analysis of condition-dependent gene expression experiments to be more useful than gene modules identified by analysis of a condition-independent data set. We have incorporated our results into the MSU Rice Genome Annotation Project database as additional expression-based annotation for 13,537 genes, 2,980 of which lack a functional annotation description. These results provide two new types of functional annotation for our database. Genes in modules are now associated with groups of genes that constitute a collective functional annotation of those modules. Additionally, the expression patterns of genes across the treatments/conditions of an expression experiment comprise a second form of useful annotation.

## Introduction

The importance of large-scale gene expression analysis in understanding gene function became apparent with the first report of genome-wide transcript expression profiling with DNA microarrays [1]. This led to the use of coexpression analyses to measure not only the physiological state of cells but also to characterize genes with no known function [2]. As more gene expression data sets became available, data from multiple experiments were combined into single analyses to functionally annotate genes based on the conditions under which they are expressed and their correlation to genes with similar expression patterns [3], [4]. In plants, numerous projects perform large-scale gene expression analyses in which coexpression networks are created. Several of these combine results from individual experiments and utilize Pearson correlation coefficients between all gene pairs [5], [6], [7], [8], [9], [10], [11] while others incorporate multiple types of data including gene transcript levels, protein-protein interactions, metabolite profiles, and predicted conserved gene interactions [6], [12], [13], [14].

A number of publicly available gene coexpression network databases have been constructed that allow researchers to query pre-constructed gene networks with a target gene(s). These databases permit the identification of correlated gene partners and visualization of a graphical display of coexpression networks with user-specified cutoff criteria including specific experiments or conditions upon which the correlation calculation is performed [5], [6], [7], [8], [11]. One confounding problem with current analysis and display methods is that coexpression networks can be very complex thereby making interpretation difficult. Although the selection of a correlation value cutoff can simplify a network by reducing the number of edges, the understanding of gene networks is still problematic [15], [16]. Due to the complexity of gene coexpression networks, various methods have been used to find the most informative relationships within correlation networks [17], [18], [19], [20], [21], [22], [23], [24].

Several research groups have identified subsets of highly correlated genes within large gene coexpression networks in *Arabidopsis thaliana* and rice (*Oryza sativa*) [14], [17], [18], [19], [21], [22], [25], [26]. Using various algorithms, these reports examine gene coexpression networks to identify subsets of genes that are more highly connected and highly correlated to each other than they are to other genes in the network. These subnetworks of genes are referred to as modules. Genes within such modules have been shown to be enriched for particular Gene Ontology (GO) categories [17], [18], [19], [22], and relationships depicted by gene modules are congruent with expected gene pathways [18], [19], [22]. Additionally, hypotheses formulated from gene coexpression modules for particular genetic pathways related to seed embyro development, chlorophyll degradation, organ development and lectin receptor kinase inhibition of seed germination have been substantiated by downstream laboratory experiments [18], [27], [28], [29].

Methods for analyzing genome-wide expression data are either condition-dependent or condition-independent depending on the selection of input data. Condition-dependent data consist of planned treatments/conditions that are designed to record transcript responses to specific physiological states. In contrast, condition-independent data are a compilation of unrelated treatments/conditions that are not designed to provide insight to a particular biological response. Most large-scale plant gene coexpression resources utilize condition-independent analyses that rely upon large compendia of gene expression data sets from independent sources [6], [7], [8], [9], [10], [13], [15], [17], [18], [19], [21], [22], [25], [26]. Such analyses are convenient because they make use of the maximal available data. However, there are potential problems with condition-independent analyses as it has been demonstrated that gene coexpression analysis with too many microarray samples can result in the loss of information [30]. Difficulty in interpreting the biological meaning of correlations in complex condition-independent data sets is a second problem with this analysis strategy. In contrast, condition-dependent analyses typically utilize a smaller, defined set of treatments or conditions that have been chosen to test a particular hypothesis or offer insight into a specific physiological condition [15], [16]. Nonetheless, both condition-independent and condition-dependent gene coexpression studies have utility. Analyses from large condition-independent data sets are likely to identify highly conserved core gene networks while smaller condition-dependent experiments offer the opportunity to recognize more narrowly defined correlations.

In this study, we have adopted a condition-dependent approach and have separately analyzed fifteen rice gene expression data sets based on the Affymetrix Gene-Chip Rice Genome Array using Weighted Gene Correlation Network Analysis (WGCNA), a network analysis method that has been widely used to identify biologically meaningful gene modules in a variety of organisms [24], [31], [32], [33], [34], [35], [36]. Additionally, we created a condition-independent data set from the same fifteen rice gene expression experiments and identified gene modules from the combined data. A comparison of the results from the two analyses suggests that while both have utility, the data analysis from individual experiments facilitates biological interpretation and is less likely to obscure uncommon but potentially informative gene coexpression modules than the combined data set. Using the condition-dependent results, we have supplemented the annotation of rice genes as 17,298 of the 40,829 protein coding genes in the MSU Rice Genome Annotation Project lack assigned functional annotation [37]. These results provide two important types of annotation. Genes included in these analyses are now associated with expression patterns across defined treatments/conditions. Additionally, genes that have been assigned to coexpression modules can be considered in the context of all other genes that are found within the same module. Both module membership and individual gene expression patterns have been incorporated as part of the annotation in the MSU Rice Genome Annotation Project database (http://rice.plantbiology.msu.edu) [37].

## Results

### Datasets Used in This Study

Publicly available rice gene expression data were downloaded from the National Center for Biotechnology Information Gene Expression Omnibus (NCBI GEO) and European Bioinformatics Institute (EBI) ArrayExpress [38], [39] in February 2010. Only data that had been generated using the Affymetrix Rice GeneChip were considered for analysis. In total, fifteen data sets were chosen for analysis in this study representing 440 arrays (Tables 1, S1, S2). The experimental conditions used to generate the data sets included biotic and abiotic stresses, cytokinin treatment, gibberellin signalling pathway mutant analysis, an extensive tissue atlas, seed germination time courses, an inflorescence and seed developmental series, and photoperiod/thermoperiod time courses [40], [41], [42], [43], [44], [45], [46], [47], [48], [49], [50]. Not all samples or treatments/conditions for each data set were included in the analyses. In a few experiments, some treatments/conditions were excluded in order to simplify the interpretation of the results. For example, only expression data for a single rice cultivar, Minghui 63, were included in the analysis of the GSE19024 tissue atlas. Also, root and leaf samples were not essential for the GSE6893 inflorescence and seed developmental series, and root and leaf samples were removed from the dataset. Some individual chips were also excluded after quality analysis (see Materials and Methods), and in two cases, this resulted in all replicates for a single treatment being discarded: shoot −Fe+P from GSE17245 and LL LDHC 124 hrs from E-MEXP-2506. Descriptions of the chips that were analyzed for each experiment in this study as well as the number of arrays and samples/treatments per experiment are provided in Tables S1 and S2.

**Table 1:** Rice gene expression data sets and analysis parameters used in this study.

Data Set^1^	Description	CV Cutoff^2^	Beta Parameter^3^	Tree Cut Parameter^3^
GSE4471	Arsenate response in roots of cultivars Azucena and Bala [40]	0.6	15	0.6
GSE6719	Cytokinin response in roots and leaves [41]	0.8	15	0.9
GSE6893^4^	Inflorescence and seed developmental series [42]	0.8	22	0.9
GSE6901	Seedlings treated with abiotic stresses [42]	0.8	15	0.8
GSE10373	*Striga hermonthica* infection time course from roots of cultivars IAC165 and Nipponbare [43]	0.6	10	0.8
GSE11025	Rice stripe virus infection of seedlings of cultivars WuYun3 and KT95	0.6	15	0.7
GSE15046	Analysis of shoots of gibberellin signalling mutants [44]	0.6	15	0.9
GSE16793	Infection by *Xanthomonas oryzae* pv. *oryzae* or by *X. oryzae* pv. *oryzicola*	0.6	15	0.9
GSE17245^5^	Iron and phosphorus interactions in shoots and roots [45]	0.6	15	0.9
GSE18361	Time course of root infection with *Magnaporthe oryzae* Guy11 [46]	0.6	30	0.9
GSE19024^6^	Tissue atlas from cultivar Minghui 63 [47]	0.8	11	0.8
GSE19239	Response of transgenic rice with maize *Rxo1* gene to infection by *Xanthomonas oryzae* pv. *oryzicola* [48]	0.6	15	0.7
E-MEXP-1766	Time course from aerobic germination of seeds [49]	0.7	15	0.7
E-MEXP-2267	Time course from anaerobic/aerobic germination of seeds [50]	0.7	15	0.9
E-MEXP-2506^7^	Thermoperiod/photoperiod time courses	0.6	7	0.9
Combined data set	Combined chips from all 15 individual experiments	0.9	4	0.95

1 Identifiers for data are from either NCBI GEO or EBI ArrayExpress.

2 Coefficient of variation cutoff used to filter averaged and normalized gene expression data.

3 Beta and tree cut parameters used during WGCNA analysis of expression data.

4 Only shoot apical meristem, developing panicle and developing seed samples were used for this analysis.

5 Shoot −Fe+P samples were removed after chip QC analysis.

6 Only data from Minghui 63 were analyzed. Expression data from Zhenshan 97 were excluded from analysis. Callus tissue samples were not included in the analysis.

7 The LL-LDHC-124 hrs sample was excluded from analysis after chip QC analysis.

Data from each experiment were analyzed individually or as a single combined data set using the WGCNA method [24]. The goals of the analyses were to identify modules of highly coexpressed genes using both methods (condition-dependent and condition-independent) and then to select the method with the most informative results for supplemental rice gene annotation. For both methods, normalized trend plots were generated for all gene modules. WGCNA analyses were assessed by the number of modules identified, the similarity of expression values for the genes within a module, and the biological interpretability of the expression patterns of the genes within modules. Although relaxation of WGCNA-required parameters would have resulted in additional genes being assigned to modules, this would have reduced the overall correlation of the genes in each module (see Materials and Methods, Table 1).

### Coexpression analyses from individual, condition-dependent experiments

**Figure 1:**
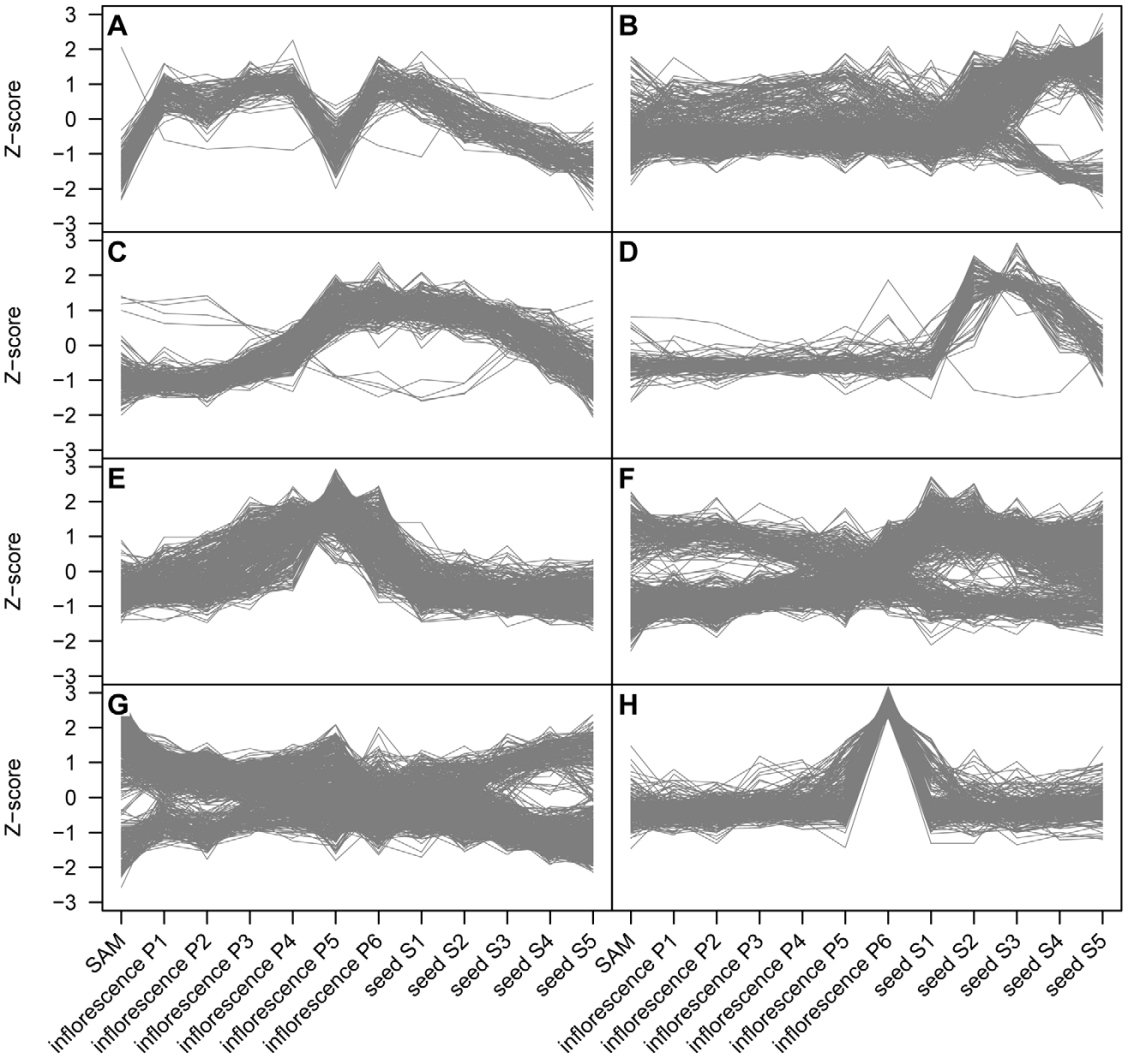
Normalized expression values of modules of genes identified from a panicle/seed developmental series. Gene expression values from a panicle and seed developmental series were processed using Weighted Gene Coexpression Network Analysis to identify modules of highly correlated genes [36], [42]. Tissues analyzed were shoot apical meristems (SAM), panicles between 0 and 3 cm long (inflorescence P1), panicles between 3 and 5 cm long (inflorescence P2), panicles between 5 and 10 cm long (inflorescence P3), panicles between 10 and 15 cm long (inflorescence P4), panicles between 15 and 20 cm long (inflorescence P5), between 22 and 30 cm long - mature pollen stage (P6), developing seed 0 to 2 days after pollination (dap; seed S1), developing seed 3 to 4 dap (seed S2), developing seed 5 to 10 dap (seed S3), developing seed 11 to 20 dap (seed S4), developing seed 21 to 29 dap (seed S5). Expression data are represented here as normalized values (Z-scores). Modules names: (A) GSE6893-black, (B) GSE6893-blue, (C) GSE6893-red, (D) GSE6893-pink, (E) GSE6893-yellow, (F) GSE6893-brown, (G) GSE6893-turquoise, (H) GSE6893-green.

**Figure 2:**
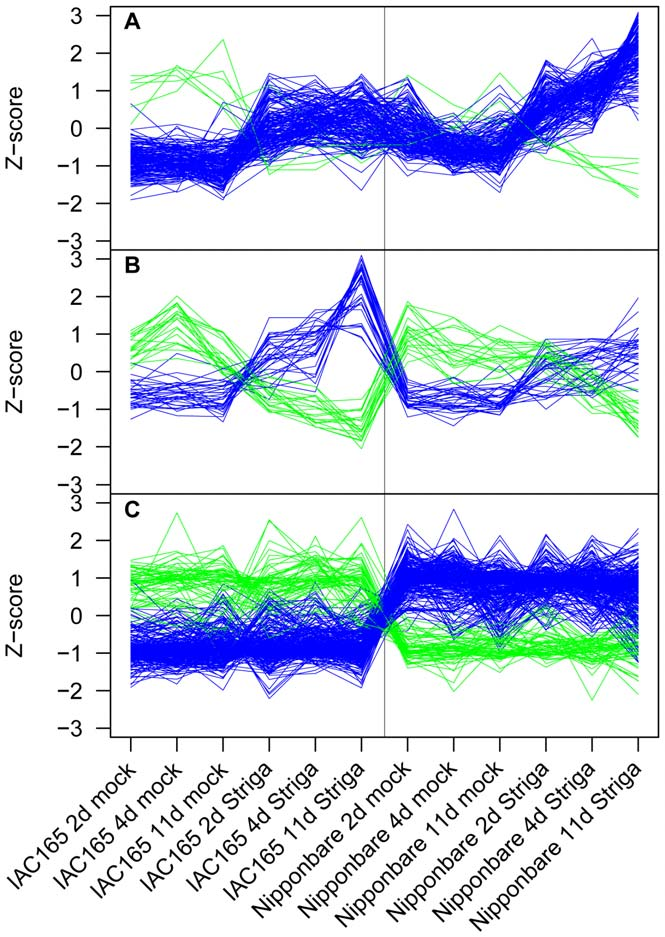
Normalized expression values of modules of genes identified from a *Striga* root infection study. Gene expression values from *Striga hermonthica* root infection time course of rice cultivars IAC165 and Nipponbare were processed using Weighted Gene Coexpression Network Analysis to identify modules of highly correlated genes [36], [43]. Expression data are represented here as normalized values (Z-scores). Two gene modules, (A) GSE10373-blue and (B) GSE10373-brown, display differential responses between genes in the two cultivars in response to infection by *S. hermonthica*. Genes from one module, (C) GSE10373-turquoise, are differentially expressed between the two rice cultivars but are not responsive to infection by *S. hermonthica*. Plots for genes that are positively correlated with each other within a module are shown in the same color. Genes within a module that are displayed in different colors are anti-correlated.

Following coefficient of variation (CV) filtering of the condition-dependent experiments, a total of 13,537 genes were retained for gene coexpression analysis in at least one experiment (range 672 to 7,478; Table S3). From all 15 experiments, 71 coexpression modules were identified containing 12,328 non-redundant genes (Table 2, Figures 1, 2, S1, S2, S3, S4, S5, S6, S7, S8, S9, S10, S11, S12, S13). The remaining 1,209 genes that passed CV filtering were not assigned to any coexpression module. The number of modules identified within an experiment varied from two to nine, and the number of genes assigned to all modules within a single experiment ranged from 567 to 4,566. Modules contained between 40 and 3,574 genes with an average module size of 405 genes. The majority of genes assigned to coexpression modules have functional annotation, but nearly one fifth (2,908) of all genes assigned to modules lack functional annotation. Transposable element (TE) related loci were included in the gene sets for these analyses, but overall, only 406 of the genes assigned to modules were TE-related (Table 2), consistent with their reduced levels of expression. While a gene can be present in only one module from a single experiment, many genes were found in multiple modules from different experiments (Table 3). In fact, most genes that had been assigned to modules were found in modules from two or more experiments, and one gene, LOC_Os11g31540, a BRASSINOSTEROID INSENSITIVE 1-associated receptor kinase 1 precursor, was found in modules from 12 different experiments (Table 3).

**Table 2:** Numbers of genes and annotation status of genes assigned to modules by two analysis methods.

	Number of Genes Analyzed^1^	Number of Modules	Genes with Functional Annotation	Genes without Functional Annotation	TE-related Genes^2^	Total Genes Assigned to Modules
**Experiment ID** 3						
GSE4471	2,613	6	1,777	672	83	2,532
GSE6719	2,802	5	2,268	478	40	2,786
GSE6893	4,231	8	2,340	600	68	3,008
GSE6901	739	3	565	131	14	710
GSE10373	672	3	395	144	28	567
GSE11025	835	4	535	176	18	729
GSE15046	1,197	6	976	190	20	1,186
GSE16793	678	2	469	93	7	569
GSE17245	4,747	5	3,679	823	64	4,566
GSE18361	1,162	3	741	227	41	1,009
GSE19024	7,478	5	1,453	435	45	1,933
GSE19239	1,990	5	1,363	499	82	1,944
E-MEXP-1766	3,704	3	2,986	605	68	3,659
E-MEXP-2267	2,421	4	1,835	441	49	2,325
E-MEXP-2506	1,816	9	844	266	97	1,207
**Non-redundant totals from individual experiments**						
	13,537	71	9,014	2,908	406	12,328
**Combined condition-independent data set** 4						
	17,320	15	7,481	2,403	193	10,077

1 Number of genes that had passed the CV filter and that were subsequently analyzed by the WGCNA method.

2 Transposable element-related genes.

3 Identifiers for data from either NCBI GEO or EBI ArrayExpress.

4 The condition-independent data set contained all gene chips used in the analyses of each of the 15 individual experiments.

**Table 3:** Number of genes assigned to modules from different experiments.

Number of experiments	Number of genes
1	5,170
2	2,768
3	1,860
4	1,212
5	698
6	374
7	149
8	64
9	25
10	7
11	0
12	1

The numbers listed only include those genes that passed the coefficient of variation filtering and were assigned to a module of highly correlated genes. Genes that passed the coefficient of variation filtering but that were unassigned to a module were excluded from this analysis.

The gene coexpression modules identified from the panicle and seed developmental series (GSE6893, [42]) are illustrative of the results that can be obtained using WGCNA analysis with coexpression data. Expression values from a total of 4,231 genes were analyzed from this experiment (Table S3). Eight modules were identified, and the number of genes per module ranged from 104 to 725 with 1,223 genes not assigned to any module. The expression patterns for each module are distinctive (Figure 1). Some modules coincide with very specific periods of growth such as anthesis (Figure 1H), middle seed development (Figure 1D) or late panicle maturation (Figure 1E). Two modules show gene expression levels that are elevated during both panicle and seed development (Figures 1A, 1C). Three modules contain genes that are both positively and negatively correlated and that have expression levels that are alternately high and low in panicles and seeds (Figures 1B, 1F, 1G).

Gene modules obtained by analysis of expression data from a pathogen response experiment (GSE10373) are shown in Figure 2 [43]. This time course experiment was performed on two rice genotypes, Nipponbare and IAC165, after two treatment conditions, mock inoculation and infection with the parasitic weed *Striga hermonthica*. Because the samples were all derived from the same tissue type (roots), fewer genes (672) passed the CV filter relative to the developmental time course that contained a variety of tissue types (Figure 1). The genes were split into three modules ranging in size from 52 to 351 (Table S3) that display either genotype by treatment responses (Figures 2A and 2B) or genotype specific expression (Figure 2C).

Enrichment analysis was performed to identify genes containing particular Pfam domains that are over-represented in these coexpression modules (Tables 4, S4). Statistically significant enrichment was observed in modules from all 15 experiments analyzed. A total of 61 modules were found to have enrichment of genes with at least one Pfam domain, and 114 Pfam domains were enriched in at least one module. A number of modules had enrichment of Pfam domains consistent with the assayed biology. For example, the GSE6893-blue module contains genes that are expressed during late seed development (Figure 1B) and enrichment of genes with seed-related cupin, protease inhibitor/seed storage/LTP family and starch synthase catalytic Pfam domains was evident (Table S4) [51], [52], [53]. Also, the GSE10373-blue, GSE16793-blue and GSE18361-blue modules have higher than expected numbers of genes with terpene synthase, WRKY DNA binding and chitinase domains, all domains that are found in genes that are known to be responsive to biotic stresses (Tables S4, S5) [54], [55], [56], [57].

**Table 4:** Number of gene coexpression modules and number of enriched Pfam domains associated with different experiments.

Experiment	Number of Modules Analyzed	Number Modules with Pfam Enrichment	Number Unique Pfam Domains Enriched within Experiment
E-MEXP-1766	3	3	24
E-MEXP-2267	4	3	19
E-MEXP-2506	9	9	31
GSE10373	3	3	8
GSE11025	4	3	4
GSE15046	6	3	14
GSE16793	2	2	9
GSE17245	5	5	25
GSE18361	3	3	26
GSE19024	5	4	21
GSE19239	5	4	11
GSE4471	6	5	13
GSE6719	5	4	14
GSE6893	8	7	35
GSE6901	3	3	7

### Coexpression analyses from combined, condition-independent experiments

A condition-independent data set was constructed by combining all data from the 15 condition-dependent experiments used above and performing coexpression analysis with WGCNA. After CV filtering 17,320 genes were used for gene module identification using WGCNA. Only 15 modules containing 10,077 genes were identified from the combined data set (Tables 2, S6). Those modules varied in size from 40 to 3,740 genes and had an average size of 671 genes. There were 7,481 non-TE related genes with functional annotation and 2,403 genes with no functional annotation assigned to modules. Enrichment analysis was also performed to identify Pfam domains that were over-represented in genes from the condition-independent coexpression modules. A total of 14 modules had enrichment of a total of 209 Pfam domains (Table S7).

**Figure 3:**
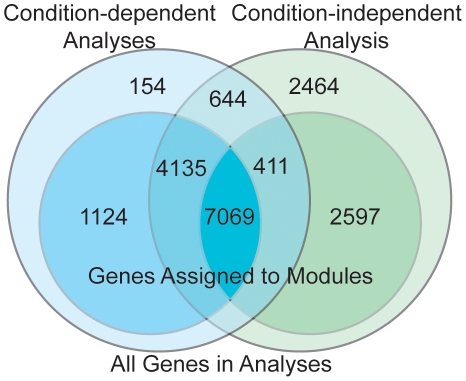
A Venn diagram showing the intersections of genes used in condition-dependent and condition-independent coexpression analyses. The blue circles on the left represent the combined results from the condition-dependent coexpression analyses. The green circles on the right represent the results from the condition-independent analysis. The inner and outer circles respectively represent the genes that were assigned to modules and those that were not assigned to modules in each of the analyses, respectively.

In combination, the condition-dependent and condition-independent analyses included 18,598 genes, of which 15,336 were assigned to at least one module from at least one analysis. Of the 12,259 genes common to both types of analysis, 11,204 were assigned to modules from the condition-dependent experiments, but only 7,480 were found in condition-independent modules. Modules from both the condition-dependent and condition-independent analyses contained a common subset of 7,069 genes. There were 5,259 genes found in at least one condition-dependent module that were not assigned to any modules from the condition-independent analysis and 3,008 genes found in a condition-independent module that were not found in any condition-dependent modules (Figure 3).

Fewer genes were assigned to gene coexpression modules from condition-independent compared to condition-dependent analyses, and there were fewer modules identified from the condition-independent analysis (Table 2). An examination of the trend plots of the condition-independent gene modules shows that some of the patterns observed in condition-dependent gene modules can be observed in condition-independent modules (e.g., Figure S9B vs. Figure S14A; Figure S9B vs. Figure S14B; Figure S13F vs. Figure S14C). Additionally, some condition-independent modules have similar gene expression patterns across a subset of conditions. Figures S14A and S14B show gene expression patterns from the green-yellow and pink modules from the condition-independent analysis, and these modules have similar patterns of gene expression across numerous samples. However, some striking expression patterns from condition-dependent modules are not easily identified in any condition-independent modules such as the anti-correlated circadian cycles in Figures S13E and S13I or the infection response expression in Figure S6A; these expression patterns may be obscured within a densely populated condition-independent module. A figure containing all gene expression trend plots for each condition-independent gene module can be downloaded from the MSU Rice Genome Annotation FTP site (ftp://ftp.plantbiology.msu.edu/pub/data/rice_gene_assoc/Figure_condition_independent_modules.pdf).

A comparison was made to identify the overlap in genes between modules from the two strategies (Table S8). Often, a high proportion of genes from individual experiment modules were assigned to a gene coexpression module from the condition-independent analysis. This is not absolute as fewer than half of the genes from some condition-dependent modules were present in the condition-independent modules. In a few cases, the majority of genes from a condition-dependent module were almost entirely contained within a single condition-independent module. However, the more common occurrence was for genes from a single condition-dependent module to be distributed between a subset of condition-independent modules, and this was the case for the modules described above in Figures S9B, S14A, S14B, which represent the GSE19024-brown module and the condition-independent green yellow and pink modules (Table S8).

### Improvement of rice gene annotation via coexpression analyses

We incorporated the results from the analyses of individual condition-dependent experiments into the MSU Rice Genome Annotation Project [37]. An overview page (http://rice.plantbiology.msu.edu/annotation_association_analysis.shtml) provides a brief description of the procedure for identifying gene coexpression modules and contains links to pages that show trend plots for the coexpression modules for each data set analyzed. Researchers can find large-scale images of the trend plots for all modules, lists of genes from each module, and files with correlation values for all genes analyzed from each data set. Search pages allow users to query the database to explore the expression patterns of genes within a single module, within a single data set or between data sets. To enhance the functional annotation of rice genes, trend plots for all genes covered in this study are now included on the gene annotation pages. For genes assigned to a module, the trend plot for the entire module is displayed. For genes not assigned to a module, the trend plot represents only the normalized expression values for that single gene across the treatments from the relevant experiment. In both cases, links to additional information about the module and/or parent data set are also provided.

## Discussion

Gene expression data have expanded the resources available for functional annotation on a gene as well as a genomic scale. In the simplest cases, such data can help to define the tissues and conditions under which a gene is expressed. Several projects have performed correlation analyses on plant gene expression data in order to identify gene associations that may imply common functions or even regulatory relationships [6], [7], [8], [9], [10], [13], [17], [18], [19], [21], [22], [25], [26]. Many of these efforts use combined expression data sets from numerous independent experiments, and the results are typically presented in terms of complex gene association networks. In some cases, these networks are further analyzed in order to identify modules of highly correlated and connected genes.

In this study, we have performed analyses on publicly available gene expression data from a diverse collection of experiments to identify gene coexpression modules. Unlike previous studies that use combined data sets from multiple rice expression experiments [7], [14], [17], [26], here we performed gene coexpression module analysis on expression data from individual experiments and compared it with results from a combined condition-independent data set. Our motivation in performing the condition-dependent analyses was to ensure that strong correlations apparent in select conditions were not lost when multiple diverse experiments are combined. The observation that of the genes common to both analyses, over 91% were assigned to at least one gene module from the condition-dependent analyses but only 61% were found in the condition-independent gene modules supports our reasoning (Figure 3). Certainly, a slight change in analysis parameters could alter the numbers of genes in modules and thus shift the percentage of genes found in modules in the two analysis approaches. However, the large number of genes in many of the condition-independent modules present challenges in biological interpretation. More importantly, the common splitting of genes within a single condition-dependent module into multiple modules in the condition-independent analysis indicates that important functional associations between genes are lost through condition-independent analysis (Table S6, Figure 3). The likely explanation for this last observation is that genes are correlated with different groups of genes within different tissues or under different physiological states. A well-defined experiment would permit the observation of one gene coexpression module, but when data from that experiment are combined with expression data from many other experiments, the correlations between the genes from that single coexpression module will be weakened and the genes in that module may be split into numerous new gene modules. Condition-independent analyses are more likely to result in gene modules with strong coexpression correlations which can obscure weaker gene coexpression relationships that occur under a subset of conditions/treatments. The obscuring effect of condition-independent expression analyses is likely to hold regardless of the algorithm or parameters used to identify gene modules. Therefore, given that our goal was to provide functional annotation to the rice gene set by identifying as many gene modules as possible, we find that the condition-dependent gene coexpression analyses are more informative.

The condition-dependent coexpression modules have been incorporated into the MSU Rice Genome Annotation Project database as an additional form of functional annotation. Of the 40,829 non-TE-related genes in the rice genome, 11,922 were assigned to at least one gene coexpression module, and 2,908 (17%) of the 17,298 rice genes that currently lack a functional description were found in at least one module. Membership in a gene module provides two distinct types of annotation to a gene. The first is association with other genes that are similarly expressed under specific conditions, and these genes may be functionally related. The second type of annotation is simply the relative pattern of expression of the gene across experimental treatments or conditions. In fact, 5,832 genes that may have been assigned to one or more coexpression modules were also found to be unassociated with any module in at least one other experiment (Table S3). The expression patterns of all genes not assigned to modules are informative as well and have been incorporated into the MSU Rice Genome Annotation Project database.

The 71 gene coexpression modules from individual experiments are diverse and will be of interest to rice researchers as these modules define sets of genes that are expressed in specific tissues or in response to various pathogen infection, abiotic stress, hormone treatments or environmental conditions (Figures 1, 2, S1, S2, S3, S4, S5, S6, S7, S8, S9, S10, S11, S12, S13). Other modules represent cultivar-specific expression differences that are apparently unrelated to experimental treatment (Figures 2C, S1D, S4A). A statistical analysis of Pfam domain enrichment of module genes also showed that many modules have higher numbers of genes with Pfam domains related to the expected physiological state of the module, suggesting functional support for those modules (Tables 4, S4). In addition to providing annotation for genes that have been assigned to coexpression modules, the modules will be useful for formulating or supporting biological hypotheses. For example, WRKY transcription factors are often associated with regulating responses to pathogen infection [58]. A number of modules identified from biotic stress experiments contain WRKY genes, and it might be hypothesized that those transcription factors regulate the expression of other genes within those modules. Also, a set of four terpene synthases and one cytochrome P450 are coexpressed in a single module from each of the *Xanthomonas*, *Magnaporthe oryzae* and *S. hermonthica* infection studies (Table S5), suggesting that these genes may be commonly expressed in response to a variety of biotic stresses. In contrast, numerous other chitinases, cytochrome P450s and terpene synthases were found in only one or two of these same gene modules suggesting that these genes are elicited by specific biotic stresses.

When performing coexpression analysis, the choice of using a combined condition-independent data set or individual condition-dependent data depends on the goal. Additionally, the choice of parameter values will affect the numbers of modules identified and the number of genes found within those modules. The coexpression modules obtained from both condition-dependent and condition-independent data analysis are likely to be biologically relevant given that Pfam domain enrichment was observed (Tables 4, S4, S7). However, for the purposes of providing annotation to rice genes, we found that the coexpression modules identified from condition-dependent data are easier to interpret as their expression patterns are generally related to a set of treatments or tissues that are functionally related. As our goal was to provide annotation that would be intuitive to interpret, we used the normalized trend plots to guide our selection of parameters. We attempted to include as many genes as possible while obtaining gene modules with trend plots that were interpretable in a biological context. With condition-dependent analyses, we observed that genes can be assigned to multiple coexpression modules in different experiments providing numerous fine-scaled annotations that are more informative than assignment of a gene to a single module in the condition-independent method. Moreover, the multiple distinct coexpression correlations that a gene has under different physiological states can be lost or difficult to observe in condition-independent gene modules. Importantly, for an annotation project, performing gene module analysis on data from individual experiments is extensible. When new expression data become available, the results can be analyzed and added to the existing annotation. With condition-independent analysis, current coexpression results would have to be discarded and replaced with the newest analysis. Some correlations could be lost in this process, and users will find such losses to be disconcerting.

We elected to use the WGCNA method to identify coexpression modules, but the general observations from our condition-dependent versus condition-independent comparison are not expected to be different if other methods are employed. This is due in large part to the fact that most coexpression network analyses rely upon gene correlation measures, and it is the combination of expression data in a condition-independent fashion that obscures relationships that are more easily observed when condition-dependent data sets are used.

## Materials and Methods

CEL files for publicly available rice expression data sets based on the Affymetrix Rice GeneChip were downloaded from either the NCBI GEO or EBI ArrayExpress [38], [39] (Table S1). Arrays from individual experiments were normalized using the liwong method as implemented in the R affy package [59], [60]. Quality tests were performed on the normalized array data using the Bioconductor arrayQualityMetrics package [61], [62], and by examining chip trees generated by the R WGCNA package [36]. Chips that were of questionable quality were discarded. A list of all CEL files that were retained from each data set is provided in Table S1.

Probe sets from the Affymetrix Rice GeneChip were mapped to the MSU Rice Genome Annotation Project gene set (release 6.1) [37]. Individual probes were aligned to representative gene models using the vmatch alignment tool (http://www.vmatch.de). Probe sets were assigned to genes if nine or more probes from the set perfectly aligned to a single gene. Probe sets that mapped to multiple genes were discarded. If two or more probe sets mapped to a single gene, the expression value for that gene was determined by averaging the signals across the probe sets. Expression values were log_2_-transformed before being processed further. Normalized and log_2_-transformed expression values were averaged across replicate chips to generate an averaged expression value for each gene from each treatment/sample. With experiment GSE19024, biological and technical replicates were available for a subset of samples, and these were treated as simple replicates for purposes of averaging.

To reduce the number of genes for the final processing, a CV (CV = μ/σ) filter was applied to the averaged expression values for a single gene across a single set of conditions/treatments (condition-dependent data) or across all combined conditions/treatments (condition-independent data) using a custom Perl script. The effect of CV filtering is to remove genes that are constitutively expressed, unexpressed or vary only modestly across experimental treatments or conditions. The CV cutoff values were determined in an *ad hoc* fashion with smaller CV values resulting in more genes passing the filter. Final CV values were chosen based on the number and quality of coexpression modules that were generated by WGCNA analysis (Table 1).

The WGCNA package for R was used to identify gene coexpression modules from the normalized, log_2_ transformed, CV filtered gene expression values [36]. Briefly, the WGCNA procedure calculates an unsigned expression Pearson's correlation matrix for all genes, transforms the correlation matrix by raising all values to a power ß, calculates a topological overlap matrix from the transformed correlation matrix, converts the topological overlap matrix into a dissimilarity matrix, creates a hierarchical cluster tree based on the dissimilarity matrix, and identifies gene coexpression modules from the hierarchical cluster tree using a dynamic tree cut procedure [24]. Unsigned correlations were used so that positively and negatively correlated genes could be grouped into the same cluster. The effect of transforming correlation values with the exponent ß is a form of soft thresholding that serves to strengthen strong correlation values while lessening but not discarding weak correlations. The use of soft thresholding is important for the topological overlap matrix calculation which measures the strength of two genes' correlation based on not just their direct correlation value but also the weighted correlations of all of their common neighbors [24], [63]. The pickSoftThreshold function in the WGCNA package was used to determine suggested ß values. However, for most of the condition-dependent analyses, an obvious ß was not identified by this method, and in all cases, several values were tested. Higher ß values result in fewer genes with strong transformed correlation values, but with smaller ß values more genes have stronger transformed correlation values [24]. Therefore, larger ß values result in fewer genes being placed in fewer modules. Smaller ß values resulted in more genes in more modules, but with smaller the ß values, more inconsistent expression patterns of genes within individual modules were observed. The condition-independent data set used a ß value that was indicated by the WGCNA pickSoftThreshold function. A range of treecut values was also tested for module detection with larger treecut values resulting in more genes being assigned to more modules. As with the CV filter value, final ß and treecut values were chosen based on the number and quality of coexpression modules identified. All other WGCNA parameters remained at their default settings. Assessment of module quality was assisted by examining trend plots of Z-score normalized expression values for all genes in a given module (Figures 1, 2, S1 to S). Custom Perl scripts were written to identify genes that were common to modules from both condition-independent and condition-dependent analyses.

Gene coexpression modules were tested for enrichment of genes containing Pfam domains that have been annotated within rice genes [37], [64]. Statistical significance for enrichment of genes containing a particular Pfam domain was assessed using the hypergeometric distribution. A Bonferroni correction was applied to an α = 0.01 when determining statistical significance of observed Pfam domain enrichment.

## Supporting Information

Figure S1 **Normalized expression values of modules of genes identified from an arsenate stress study.** Gene expression values from roots of rice cultivars Azucena and Bala grown in 0 ppm or 1 ppm AsO_4_ were processed using Weighted Gene Coexpression Network Analysis to identify modules of highly correlated genes [36], [40]. Expression data are represented here as normalized values (Z-scores). Genes up- or down-regulated in response to AsO_4_ in both Azucena and Bala rice: (A) GSE4471-blue and (B) GSE4471-brown modules. Genes differentially regulated in Azucena and Bala and responsive to AsO_4_: (C) GSE4471-green and (D) GSE4471-red modules. Genes differentially regulated in Azucena and Bala but not strongly responsive to AsO_4_: (E) GSE4471-turquoise module. Genes responsive to AsO_4_ in Azucena but not strongly responsive in Bala: (F) GSE4471-yellow module.
(EPS)

Figure S2 **Normalized expression values of modules of genes from roots and leaves in response to zeatin.** Gene expression values from roots and leaves 30 and 120 min after zeatin application were processed using Weighted Gene Coexpression Network Analysis to identify modules of highly correlated genes [36], [41]. Expression data are represented here as normalized values (Z-scores). Genes responsive to zeatin treatment in roots, (A) GSE6719-blue module. Genes responsive to zeatin treatment in both roots and leaves, (B) GSE6719-brown module. Genes from leaves responsive to zeatin treatement, (C) GSE6719-green module. Genes differentially regulated in roots and leaves and also possibly regulated by zeatin, (D) GSE6719-turquoise module. Genes more strongly responsive to zeatin in roots compared to leaves, (E) GSE6719-yellow module.
(EPS)

Figure S3 **Normalized expression values of modules of genes from seedlings in response to abiotic stresses.** Gene expression values from seedlings 3 hours after stress treatments were processed using Weighted Gene Coexpression Network Analysis to identify modules of highly correlated genes [36], [42]. Expression data are represented here as normalized values (Z-scores). Genes responsive to salt stress, (A) GSE6901-blue module. Genes responsive to cold treatment, (B) GSE6901-brown module. Genes differentially regulated by drought and salt treatments, (C) GSE6901-turquoise module.
(EPS)

Figure S4 **Normalized expression values of modules of genes identified after rice stripe virus infection.** Gene expression values after infection with rice stripe virus (RSV) of rice cultivars WuYun3 and KT95 were processed using Weighted Gene Coexpression Network Analysis to identify modules of highly correlated genes [36]. Expression data are represented here as normalized values (Z-scores). Genes differentially expressed in WuYun3 and KT95 but not strongly regulated by RSV infection, (A) GSE11025-blue module. Genes differentially responsive to RSV infection, (B) GSE11025-brown and (C) GSE11025-turquoise modules. Genes differentially regulated by RSV infection in cultivar KT95 but not affected in cultivar WuYun3, (D) GSE11025-yellow module.
(EPS)

Figure S5 **Normalized expression values of modules of genes expressed in gibberellin signalling mutants.** Gene expression values from shoots from wild type (Taichung 65) and three gibberellin signalling mutants (*gid1-3*, *gid2-1*, *slr1*) were processed using Weighted Gene Coexpression Network Analysis to identify modules of highly correlated genes [36], [44]. Expression data are represented here as normalized values (Z-scores). Genes differetially regulated in gibberellin signalling mutants compared to wild type rice, (A) GSE15046-blue module. Genes differentially regulated in *gid1-3* mutant only, (B) GSE15046-brown module. Genes differentially expressed in *gid1-3* and *gid2-1* mutants (C) GSE15046-green module. Genes differentially expressed in mutant plants compared to wild type rice, (D) GSE15046-red module. Genes differentially expressed in wild type and *gid1-3* plants compared to *gid2-1* and *slr1* mutants, (E) GSE15046-turquoise module. Genes differentially expressed in mutant plants compared to wild type rice, (F) GSE15046-yellow module.
(EPS)

Figure S6 **Normalized expression values of modules of genes identified after bacterial infection.** Time course of gene expression values after infection with *Xanthomonas oryzae* pv. *oryzae*, *Xanthomonas oryzae* pv. *oryzicola* or mock infection were processed using Weighted Gene Coexpression Network Analysis to identify modules of highly correlated genes [36]. Expression data are represented here as normalized values (Z-scores). Genes differentially expressed after infection with peak response after 96 hours, (A) GSE16793-blue module. Genes differentially expressed after infection with major response after 8 hours, (B) GSE16793-turquoise module.
(EPS)

Figure S7 **Normalized expression values of modules of genes from roots and shoots after Fe and P treatments.** Gene expression values from 10 day old seedlings grown with or without Fe and/or P were processed using Weighted Gene Coexpression Network Analysis to identify modules of highly correlated genes [36], [45]. Expression data are represented here as normalized values (Z-scores). Genes differentially expressed in roots in response to −Fe and +P, (A) GSE17245-blue module. Genes differentially expressed in shoots in response to +F and +P, (B) GSE17245-brown module. Genes differentially expressed in response to the presence/absence of P, (C) GSE17245-green module. Genes differentially regulated in roots and shoots, (D) GSE17245-turquoise module. Genes differentially regulated in roots in response to Fe or P depravation, (E) GSE17245-yellow module.
(EPS)

Figure S8 **Normalized expression values of modules of genes identified after fungal infection.** Time course of gene expression values after infection with *Magnaporthe oryzae* strain Guy11 or mock infection were processed using Weighted Gene Coexpression Network Analysis to identify modules of highly correlated genes [36], [46]. Expression data are represented here as normalized values (Z-scores). Genes differentially expressed in response to pathogen and mock infections, (A) GSE18361-blue module. Genes differentially expressed 2 days after mock infection, (B) GSE18361-brown module. Genes differentially expressed 2 days after pathogen infection, (C) GSE18361-turquoise module.
(EPS)

Figure S9 **Normalized expression values of modules of genes from a rice tissue survey.** Gene expression values from various tissues were processed using Weighted Gene Coexpression Network Analysis to identify modules of highly correlated genes [36], [47]. Tissues sampled: germinating seed harvested 72 hour post imbibition (germinating seed); light and dark grown plumules harvested 48 h after germination (plumule 1, plumule 2); light and dark grown radicles harvested 48 h after germination (radicle 1, radicle 2); 3 day old seedling (seedling 1); trefoil stage seedling (seedling 2); less than 1 mm panicle (panicle 1); 3 to 5 mm panicle (panicle 2); 10 to 15 mm panicle (panicle 3); 40 to 50 mm panicle (panicle 4); heading panicle (panicle 5); palea/lemma 1 day before flowering (palea/lemma); stamen 1 day before flowering (stamen 1); spikelet 3 days post anthesis (spikelet); endosperm 7 days post anthesis (endosperm 1); endosperm 14 days post anthesis (endosperm 2); endosperm 21 days post anthesis (endosperm 3); shoot of seedling with three tillers (shoot); roots of seedling with three tillers (root); sheath tissues from plants with panicles less than 1 mm (sheath 1); sheath tissues from plants with panicles between 40 and 50 mm (sheath 2); leaf tissues from plants with panicles less than 1 mm (leaf 1); leaf tissues from plants with panicles between 40 and 50 mm (leaf 2); leaf tissues 5 days before heading (leaf 3); leaf tissues 14 days post anthesis (leaf 4); stem tissue 5 days before flowering (stem 1); stem tissue 14 days post anthesis (stem 2). Expression data are represented here as normalized values (Z-scores). Genes expressed in shoots, mature panicles, leaf sheaths and leaf blades, (A) GSE19024-blue module. Genes expressed in spikelets and seed tissues, (B) GSE19024-brown module. Genes expressed in young and mature root tissues, (C) GSE19024-green module. Genes expressed in mature panicles and stamens, (D) GSE19024-turquoise module. Genes expressed in germinating seedling tissues, developing panicles, spikelets, shoots, roots and mature stems, (E) GSE19024-yellow module.
(EPS)

Figure S10 **Normalized expression values of modules of genes from** ***Rxo1*** **transgenic rice after bacterial infection.** Gene expression values from wild type and transgenic rice containing the maize *Rxo1* resistance gene after infection with *Xanthomonas oryzae* pv. *oryzicola* or mock infection were processed using Weighted Gene Coexpression Network Analysis to identify modules of highly correlated genes [36], [48]. Expression data are represented here as normalized values (Z-scores). Genes differentially expressed in wild type rice in response to *X. oryzae* pv. *oryzicola* (XOO) infection, (A) GSE19239-blue module. Genes differentially expressed in mock-infected wild type rice compared to XOO infected wild type or *Rxo1* transgenic rice, (B) GSE19239-brown module. Genes responsive to XOO infection in *Rxo1* transgenic rice, (C) GSE19239-green module. Genes differentially expressed in XOO infected or mock-infected wild type rice compared to *Rxo1* transgenic rice, (D) GSE19239-turquoise module. Genes responsive to XOO infection in *Rxo1* transgenic rice but not differentially regulated in wild type rice in response to infection, (E) GSE19239-yellow module.
(EPS)

Figure S11 **Normalized expression values of modules of genes during aerobic germination.** Time course of gene expression values during aerobic germination were processed using Weighted Gene Coexpression Network Analysis to identify modules of highly correlated genes [36], [49]. Expression data are represented here as normalized values (Z-scores). Genes with expression peaking between 1 and 3 hours after imbibition, (A) E-MEXP-1766-blue module. Genes with expression peaking after 3 hours of imbibition, (B) E-MEXP-1766-brown module. Genes differentially expressed early or late during aerobic germination, (C) E-MEXP-1766-turquoise module.
(EPS)

Figure S12 **Normalized expression values of modules of genes during anaerobic and aerobic germination.** Time course of gene expression values during anaerobic and aerobic germination were processed using Weighted Gene Coexpression Network Analysis to identify modules of highly correlated genes [36], [50]. Rice seed was germinated aerobically, anaerobically, aerobically for 24 hours followed by anaerobic conditions or anaerobically for 24 hours followed by aerobic conditions. Expression data are represented here as normalized values (Z-scores). Genes differentially expressed in aerobic and anaerobic conditions, (A) E-MEXP-2267-blue and (B) E-MEXP-2267-brown modules. Genes differentially expressed during early anaerobic germination, (C) E-MEXP-2267-turquoise and (D) E-MEXP-2267-yellow modules.
(EPS)

Figure S13 **Normalized expression values of modules of genes during photo- and thermo-periods.** Time course of gene expression values in rice shoots during photo- and thermo-periods were processed using Weighted Gene Coexpression Network Analysis to identify modules of highly correlated genes [36]. Shoots of rice plants were harvested every four hours. Treatments consisted of photo- and thermo-periods or constant light or temperature conditions: photocycles (LDHH), 12 hours light (L)/12 hours dark (D) at a constant temperature (31C; HH); photo/thermocycles (LDHC): 12 hours light (L) /12 hours dark (D) with a high day temperature (31C) and a low night temperature (20C); thermocycles (LLHC): continuous light (LL) with 12 hours high/12 hours low temperature (31C, day; 20C, night); and an initial 48 hours of continuous light followed by cycling photo- and/or thermo-periods (LL LDHC, LL LDHH, LL LLHC). Expression data are represented here as normalized values (Z-scores). Genes without distinct oscillation patterns under any conditions, (A) E-MEXP-2506-black. Genes differentially expressed in response to LL LDHH treatment, (B) E-MEXP-2506-blue module. Genes that cycle after an initial constant light entrainment (LL LDHC, LL LDHH, LL LLHC), (C) E-MEXP-2506-brown module. Genes that cycle the most strongly after an initial constant light entrainment (LL LDHC, LL LDHH, LL LLHC), (D) E-MEXP-2506-green, Genes that cycle during the first 48 hours of a photo- or thermo-period, (E) E-MEXP-2506-magenta module. Genes that are that require a constant light or temperature conditions, (F) E-MEXP-2506-pink and (G) E-MEXP-2506-red modules. Genes without distinct oscillation patterns under any conditions, (H) E-MEXP-2506- turquoise. Genes that cycle during the first 48 hours of a photo- or thermo-period, (I) E-MEXP-2506-yellow module.
(EPS)

Figure S14 **Normalized expression values of gene modules identified by coexpression analysis of 15 combined expression experiments.** The combined data set used the fifteen expression experiments described in the Materials and Methods section. The experimental conditions/treatments are described in the legends for Figures 1, 2 and S1 to S: green-yellow (A), pink (B) and midnight-blue (C).
(EPS)

Table S1 **Descriptions of CEL files used for coexpression analyses.**
(XLS)

Table S2 **Description of numbers of arrays used for each sample from each expression data set analyzed for coexpression analysis.**
(XLS)

Table S3 **List of module names, member genes and gene functional annotations from condition-dependent network analyses.**
(XLS)

Table S4 **Pfam domain enrichment within condition-dependent gene coexpression modules.**
(XLS)

Table S5 **Membership of genes in three coexpression modules enriched in Pfam domains for cytochrome P450, chitinase and terpene synthases.**
(XLS)

Table S6 **List of module names, member genes and gene functional annotations from condition-independent network analysis.**
(XLS)

Table S7 **Pfam domain enrichment within condition-independent gene coexpression modules.**
(XLS)

Table S8 **Overlap of genes between condition-dependent gene modules and condition-independent gene modules.**
(XLS)
